# M. Dubois on the Auscultatory Signs of Pregnancy

**Published:** 1842-04

**Authors:** 


*M. Dubois on the Auscultatory Signs of Pregnancy.*—The uterine *souffle* is usually perceptible about the fourteenth or fifteenth week of pregnancy; the period at which it may be first heard, being no doubt dependent upon the amount of development of the uterus and its elevation above the os pubis. The point at which it is most frequently audible is towards the middle of the height of the uterus on its anterior or lateral (generally the left side) part. In this respect M. *Dubois* differs from M. *Naegele*, who states that the common situation of the uterine blowing sound is one of the inguinal regions, extending thence upwards. In most cases, the space over which it may be heard is limited to a circle of two or three inches in circumference. A curious circumstance connected with this sound is the occasional changeableness of its situation; on one day it is inaudible at a spot where it had been distinctly heard the day before, and *vice versa*.

Obstetrical auscultators should be aware of this fact; else they will be apt to be perplexed in some cases. We may mention likewise that the uterine *souffle* varies much at different times in its loudness and distinctness, being one day scarcely audible, and on the next, perhaps, very distinct.

That the development of this sound is somehow dependent upon the circulation of the blood through the uterus, appears from the fact that it is always much enfeebled, or even altogether suspended, by the contractions of the organ during parturition—a fact which abundantly proves that the sound cannot proceed from the pressure of the gravid uterus on the iliac arteries, as some writers have alleged. The striking resemblance of the uterine *bruit* to that perceived in erectile tumors, and in aneurismal varices, confirms the above opinion. M. *Dubois* objects to the appellation of placentary or utero-placentary being applied to this blowing sound, for the reason that, although its locality most frequently corresponds with the attachment of the placenta, it continues to be audible for some time after the expulsion of this body, and in other cases after the death of the fœtus.

The other sound, that of the fœtal heart, is a still more decisive sign of pregnancy: the number of the pulsations varies, according to the experience of M. *Dubois*, from 135 to 150.* This tictac sound is usually most distinctly perceived on the anterior part of the abdomen somewhat to the left side: it is rarely audible before the completion of four, or four and a half months of pregnancy.—*Medico-Chir. Review, Jan.* 1842, *p.* 197.

* It is a curious circumstance, that the late Dr. Hamilton, so long the professor of midwifery in the University of Edinburgh, most stoutly maintained that the pulse of the child in utero, and also after birth until breathing commenced, seldom exceeded 60 or 70 pulsations in the minute. In our notice of his last work, in the number of the Medico-Chirurgical Review for July 1836, we questioned the accuracy of the doctor's assertions, and incurred in consequence his wrathful criticism. Our readers may find it worth their while to revert to his pamphlet, and our rejoinder, in the number of this Review for January 1837.—*Rev.*

